# Research Progress on the Immunological Correlation Between Papillary Thyroid Carcinoma and Hashimoto's Thyroiditis

**DOI:** 10.1155/jimr/7192808

**Published:** 2025-04-23

**Authors:** Digui Fang, Limei Zhou, Biao Zheng

**Affiliations:** Department of Thyroid and Parathyroid Surgery, The First Dongguan Affiliated Hospital, Guangdong Medical University, Dongguan, China

## Abstract

In recent years, a growing body of evidence has suggested a correlation between Hashimoto's thyroiditis (HT) and the onset and progression of papillary thyroid carcinoma (PTC). However, the mechanism underlying the relationship between HT and PTC remains incompletely understood. This review discusses the literature on the correlation between PTC and HT and summarizes the research concerning the immunological interplay between these two conditions. It also delves into tumor-associated cells (such as CD8+ T cells), tumor-associated macrophages (TAMs), regulatory T cells (Tregs), and cancer-associated fibroblasts (CAFs), alongside other tumor-associated factors, including interleukins (ILs), interferon-gamma (IFN-*γ*), tumor necrosis factor-alpha (TNF-*α*), cyclooxygenase-2 (COX-2), inducible nitric oxide synthase (iNOS), and hypoxia-inducible factor-1 (HIF-1), highlighting their roles in the interaction between PTC and HT. We also explore the strategic direction of immunotherapy in thyroid malignancies, particularly PTC with HT, and propose novel targeted immunotherapies for advanced thyroid cancer.

## 1. Introduction

According to the 2020 Global Cancer Incidence and Mortality Database established by the World Health Organization International Agency for Research on Cancer, thyroid cancer is the ninth most common malignancy worldwide [1]. Papillary thyroid carcinoma (PTC) has the highest incidence of all the thyroid malignancies [2]. PTC accounts for more than 80% of thyroid cancers [3, 4], is the most common type of thyroid cancer in adults, particularly in children, and frequently occurs in women aged 30–45 years. PTC is well differentiated and less malignant than other types of thyroid cancer. Although some lesions are multicentric, about one third involve both lobes of the thyroid gland; in these cases, cervical lymph node metastasis occurs earlier, but the prognosis remains favorable. Hashimoto's thyroiditis (HT) is one of the most prevalent autoimmune disorders, with its incidence varying across different regions and socioeconomic contexts. Overall, the prevalence in women ranges from 4.8% to 25.8%, while in men, it ranges from 0.9% to 7.9% [5, 6]. The disease is characterized by the presence of thyroid-specific autoantibodies, thyroid enlargement, substantial lymphocytic infiltration, and the presence of thyroid antigen-specific antibodies [7]. Cellular immunity and humoral immunity play a key role in the occurrence and development of this disease. Clinically, HT is characterized by systemic manifestations as a result of thyroid injury and progression to primary hypothyroidism. Dailey et al. [8] first proposed an association between HT and PTC in 1995. Since then, this relationship has been the focus of considerable research and controversy. In recent years, concomitant existence of these two diseases has become increasingly common, but the heterogeneity of the tumor microenvironment (TME) in PTC remains unclear. The relationship between PTC and HT remains a subject of controversy. Existing literature [9, 10] suggests that HT may elevate the incidence of differentiated thyroid cancer (DTC). However, patients with DTC associated with HT often exhibit a more favorable prognosis compared to those with non-Hashimoto's thyroid cancer. Most researchers believe that there is a relationship between chronic inflammation and malignant transformation of the thyroid gland and that inflammation creates a microenvironment conducive to tumorigenesis [9, 11, 12]. Immune cells in the TME secrete pro-inflammatory cytokines and chemokines that promote proliferation of tumor cells [13]. HT is the most common of these conditions and is considered to be a potential risk factor for PTC. However, PTC cases with concurrent HT have a relatively good prognosis, including less extrathyroid elongation rate, lymph node metastasis rate, BRAFV600E gene mutation, and lower recurrence rate [14]. Relevant studies have elucidated the shared pathogenic mechanisms underlying HT and PTC, encompassing aspects of immunity, inflammation, endocrinology, iodine deficiency, and radiation exposure [15–17]. This review article endeavors to comprehensively explore the immunological interplay between HT and PTC.

## 2. Correlation Between the Clinicopathological Features of PTC and Those of HT

**Figure 1:**
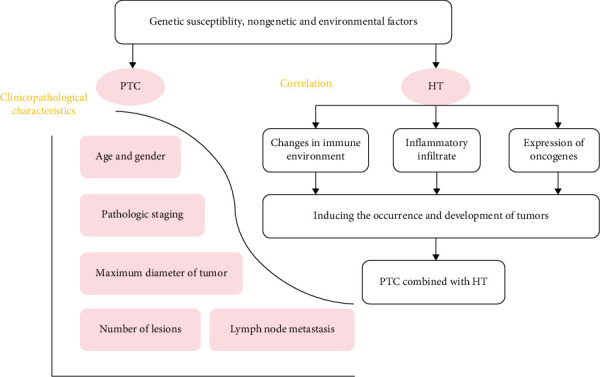
Clinicopathological characteristics of PTC and HT.

The clinicopathological (Table 1) profile of PTC encompasses various factors, including age, sex, preoperative thyroid function, antibodies (i.e., TT3, TT4, FT3, FT4, thyroid-stimulating hormone, and thyroid peroxidase antibodies), pathological stage, maximum tumor diameter, capsular and extracapsular invasion, number of lesions, and lymph node metastasis (Figure 1). PTC is the most prevalent type of thyroid cancer and is characterized by distinct pathological features. Notably, nuclear attributes, such as ground-glass nuclei, a visible nuclear sulcus, and intranuclear pseudoinclusion bodies, play an important role in the diagnosis of PTC [28]. In clinical practice, PTC predominantly affects female adolescents, grows slowly, has a propensity for late distant metastasis and typically presents at an early TNM stage. This tumor is predominantly unilateral, occurring in the left or right thyroid lobes or isthmus, and presents primarily as a solitary lesion, although multiple lesions are sometimes observed. PTC has a high incidence of cervical lymph node metastasis, with early onset, extensive distribution, gradual progression, and cystic changes. Compared to other thyroid malignancies, PTC typically exhibits a favorable prognosis and a high postoperative survival rate [23, 24]. However, unlike patients with isolated PTC, those who have PTC concomitant with HT tend to be younger, are more likely to be female and have a smaller tumor diameter and typically have elevated serum thyroglobulin antibody, thyroid peroxidase antibody, and thyroid-stimulating hormone levels [18]. Kwak et al. [19] found that the BRAF (V600E) mutation rate was lower in patients with both PTC and HT than in those with PTC alone. Given the association of BRAF mutation with the aggressiveness and progression of PTC, this finding suggested a potentially better prognosis for patients with both PTC and HT. Furthermore, in terms of pathological staging, the TNM stage was earlier in cases of concomitant PTC and HT than in those with PTC alone. Furthermore, the incidence of invasion of the thyroid capsule was lower in PTC with HT than in isolated PTC. Kim et al. [21] performed a multivariate logistic analysis with adjustment for various factors, including age, sex, tumor size, and multifocal lesions, and found an inverse association of concomitant PTC and HT with central lymph node metastasis. Similarly, Liang et al. [20] identified that HT had a limiting effect on tumor volume expansion and reduced the rate of lymph node metastasis. Although PTC tumors tend to be smaller and have a lower lymph node metastasis rate, they are more likely to be multifocal. Consequently, the prevailing consensus among researchers is that total thyroidectomy is the preferred surgical approach for patients with PTC who also have HT.

PTC usually has a favorable prognosis, with a 10-year relative survival rate of approximately 93% [25]. However, this encouraging outlook may be impacted by several factors, including the patient's age at the time of diagnosis and surgery, tumor size, TNM stage, presence of cervical lymph node or distant metastases, external thyroid extension, extent of thyroidectomy, and surgical margin status [24]. Emerging literature underscores the protective role of HT in patients with PTC. Kashima et al. [26] presented compelling evidence of disparities in outcomes between patients with PTC according to whether or not they had concomitant HT. Specifically, they observed a cancer-specific mortality rate of only 0.7% and a remarkable relapse-free survival rate of 95% among patients with both PTC and HT [26]. In contrast, patients with PTC alone had a higher mortality rate of 5% and a comparatively lower relapse-free survival rate of 85%. These findings strongly suggest a positive correlation between HT and overall survival in patients with PTC, suggesting that PTC with HT has a better prognosis.

## 3. The Tumor–Immune Microenvironment (TIME) in the Development of PTC

**Figure 2:**
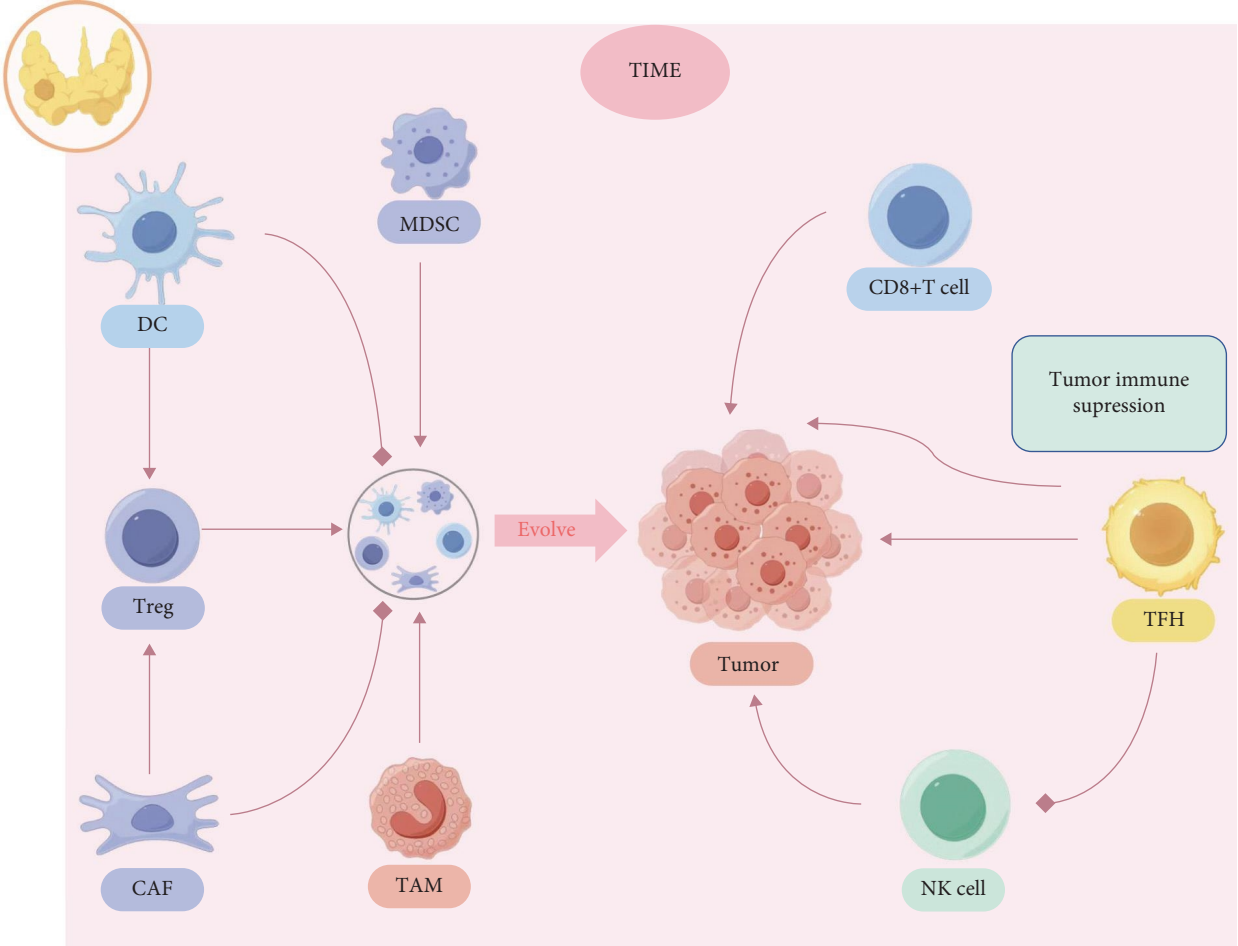
Schematic representation of cellular interactions shaping the PTC tumor microenvironment.

In solid tumors, there is a complex interplay between the TME and tumor cells. In the TME, there is a dynamic relationship between tumor initiation, progression, and metastasis within the internal and external milieu of tumor cells (Figure 2). This symbiotic relationship is characterized by mutual dependence, resistance, struggle, and promotion. Tumor cells within the TME communicate with neighboring cells in a bidirectional manner via the circulatory and lymphatic systems, thereby having a profound influence on tumorigenesis [29]. Similarly, nonmalignant cells within the TME have an important role across all stages of carcinogenesis by promoting uncontrolled cell proliferation. The interplay between malignant and nonmalignant cells within the TME has a significant impact on the development and progression of cancer [30]. Comprising a diverse array of cellular constituents, the TME includes fibroblasts, endothelial cells, immune cells, adipocytes, and extracellular matrix components [31]. Emerging evidence has highlighted a discernible association between occurrence and progression of cancer and autoimmune disorders [32]. As far back as 1863, Virchow postulated a close interconnection between the immune response and cancer [33]. In 2002, Dunn et al. [34] attempted to elucidate the intricate nexus between immunity and cancer by advancing the immunoediting hypothesis, which delineates three sequential stages of immune–tumor interaction, namely, elimination, equilibrium, and escape. The correlation between PTC and the immune system remains a focal point of research and investigation.

During the onset and progression of PTC, the human immune system undergoes augmentation, yet various immune cells assume distinct roles within the TIME. For example, M2 macrophages, regulatory T cells (Tregs), bone marrow-derived suppressor cells (BMDSCs), neutrophils, dendritic cells (DCs), cancer-associated fibroblasts (CAFs), mast cells (MCs), and M0 macrophages have tumor-promoting effects [14, 35–40]. Conversely, M1 macrophages, cytotoxic T lymphocytes (CD8 + T cells), B cells, natural killer (NK) cells, and follicular helper T (Tfh) cells have antitumor effects [41]. Various immune cells have distinct roles in tumor progression. Among the myeloid cells, myeloid-derived suppressor cells (MDSCs) have potent immunosuppressive activity [37] that is correlated with an adverse prognosis. Tumor-associated macrophages (TAMs) facilitate metastasis of PTC cells via secretion of CXCL8/interleukin (IL)-8 and paracrine interaction with CXCR1/2 [42]. Among the subtypes, M0 and M2 macrophages augment tumor invasion, whereas M1 macrophages aid in inhibition of tumor growth and angiogenesis, enhancing sensitivity to chemotherapeutic agents [43]. CAFs contribute to formation and organization of the extracellular matrix of the tumor, infiltrating the TME to influence tumor initiation, inflammation, metabolism, metastasis, drug response, and immune surveillance. Their impact on thyroid cancer is multifaceted and intricate. DCs contribute to immune function via antigen presentation and secretion of cytokines. Within the TME, DCs show diminished expression of co-stimulatory molecules and heightened levels of regulatory factors, which lead to changes in antigen presentation. Various insults can impair the function of DCs, resulting in local immunosuppression within the TME [38]. Neutrophils, in the context of PTC, may have modulated pro-inflammatory activity and express factors conducive to tumor progression. NK cells have cytotoxic activity and are important in cancer immune surveillance [44]. However, in PTC, the antitumor activity of NK cells is hindered by the immunosuppressive milieu. Further investigation of the therapeutic potential of NK cells in thyroid malignancies is warranted. The prognosis of PTC often improves with infiltration of lymphocytes, including CD8 + T cells and TFH cells. Conversely, Tregs correlate with a poorer prognosis [45], promoting progression of PTC and lymph node metastasis by interfering with the immune response [46]. MCs, as tissue-resident immune cells, can influence tumor growth, but their impact varies across different stages of tumor development [47]. The interplay of immune cells, along with a myriad cytokines and chemokines, shapes the intricate immune microenvironment in the thyroid gland and has a number of diverse roles in tumor development.

## 4. Effect of HT on the Immune Microenvironment in PTC

The incidence of HT continues to rise annually, with rates ranging from 4.8% to 25.8% in women and 0.9% to 7.9% in men [5, 6]. The onset of HT is closely linked with autoantibodies associated with lymphocyte infiltration, which increases the risk of PTC [22]. Within the microenvironment of HT, various immunoactive cells infiltrate alongside factors that include chemokines, cytokines, and growth factors and are essential in cell transformation and tumor progression [7].

### 4.1. Effect of Tumor-Infiltrating T Lymphocytes on the TIME

#### 4.1.1. Effect of Cytotoxic T Lymphocytes on the TIME

**Figure 3:**
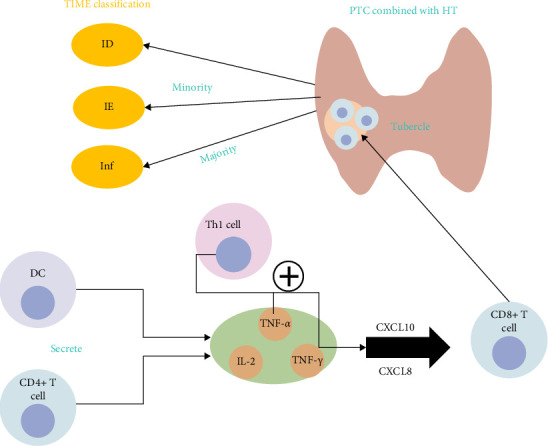
The influence of cytotoxic T lymphocytes on the TIME.

Research in thyroid cancer has revealed that Th1 lymphocytes can stimulate production of interferon-gamma (IFN-*γ*) and tumor necrosis factor-alpha (TNF-*α*), leading to release of CXCL10 and CXCL8. This cascade in turn promotes release of cytokines within the TME, thereby influencing tumor progression [48]. This phenomenon is intricately linked to the immune response observed in HT (Figure 3). The immune response in HT includes CD4+ T cells, CD8+ T cells, DCs, and Th17 cells. During the immune response, DCs and CD4+ T cells secrete IL-2, TNF-*α*, and TNF-*γ*, which have an important role in activation of CD8+ T cells [49]. Sulaieva et al. [13] observed that HT facilitates recruitment of T lymphocytes to the thyroid gland, including CD4+ T cells, CD8+ T cells, and Th17 cells [50]. The proportion and spatial distribution of CD8+ T cells at TIME are important determinants of tumor progression, that is, the number and proportion of CD8+ T cells in PTC patients combined with HT is significantly higher than that in PTC patients alone [51]. An abundance of CD8+ T cells indicates activation of an antitumor response. Co-occurrence of PTC and HT can enhance activation of the antitumor immune response, influencing the cancer immune cycle within the TIME. Based on the heterogeneous spatial distribution of CD8+ T cells, the TIME can be categorized into the following three immunophenotypes: an immunodeficiency type, characterized by a low number of lymphocytes in and around the tumor; an immunosuppressive type, with evident peritumoral infiltration, but a small number of intratumor lymphocytes, indicating limited lymphocyte infiltration into the tumor stroma; and an immunoinflammatory type, characterized by extensive lymphocyte infiltration within the tumor [13]. In the context of PTC, the presence of HT correlates predominantly with the immunoinflammatory phenotype. Within the TIME of PTC, instances where PTC coexists with HT are predominantly the immunoinflammatory phenotype, while isolated PTC cases tend to have the immunodeficiency or immunosuppressive phenotype. Notably, the prognosis of PTC appears to be more favorable for the immunoinflammatory phenotype than for the other phenotypes. The underlying reason for this difference is likely multifactorial and there is a need for further investigation of the specific mechanisms involved.

#### 4.1.2. Effect of Tregs on the TIME

Tregs (Figure 1) are a specialized subset of T lymphocytes primarily responsible for maintaining immune homeostasis and tolerance, thus, protecting the host from autoimmune diseases. However, in the context of malignancy, they can hinder immune surveillance [52]. Tregs are characterized by the expression of CD4, CD25, cytotoxic T-lymphocyte antigen-4 (CTLA-4), and the Forkhead/winged helix transcription factor (FoxP3), leading some researchers to designate them as CD4^+^CD25^+^CTLA-4^+^FoxP3^+^ T lymphocytes [36]. This discussion primarily focuses on the impact of Tregs within the TIME. Tregs can promote tumor progression and induce apoptosis by modulating cellular and molecular networks [46, 53–55]. Togashi et al. [45] proposed that Tregs exert their immunosuppressive effects through various cellular and humoral mechanisms: CTLA-4 suppresses antigen-presenting cells (APCs), consumes L-2, and produces relevant immunosuppressive factors. They also noted that programmed cell death protein-1 (PD-1) serves as a negative regulator between Tregs and effector T cells, with its expression being indicative of Treg suppressive function. The upregulation of FoxP3 levels characterizes Treg-mediated suppression of antitumor immune responses within the TIME [55]. FoxP3 not only regulates the suppressive activity of Tregs but also serves as a critical marker for their development and function [56]. In a related study, Liu et al. [27] investigated the expression of FoxP3 in simple PTC, PTC with HT, and multinodular goiter (MNG). Their results indicated that FoxP3 expression was absent in MNG tissues, while a substantial presence of tumor-infiltrating FoxP3^+^ Tregs was observed in primary PTC and lymph node tissues. Notably, the proportion of FoxP3^+^ Tregs was significantly reduced in PTC tissues combined with HT. The authors concluded that an increased percentage of FoxP3^+^ Tregs in PTC tissues compared to FoxP3^+^ Tregs may be associated with tumor aggressiveness, suggesting that patients with HT concomitant with PTC may have better prognoses than those with simple PTC. Although the prognostic impact of Tregs varies across different cancer types, the majority of studies report a positive correlation between FoxP3^+^ Tregs and poor prognosis in various malignancies. As Tregs continue to be a focal point of research, numerous scholars are exploring their potential as novel therapeutic targets. Future prospective and longitudinal studies will be essential [36, 52, 53].

### 4.2. Effect of TAMs on the TIME

**Figure 4:**
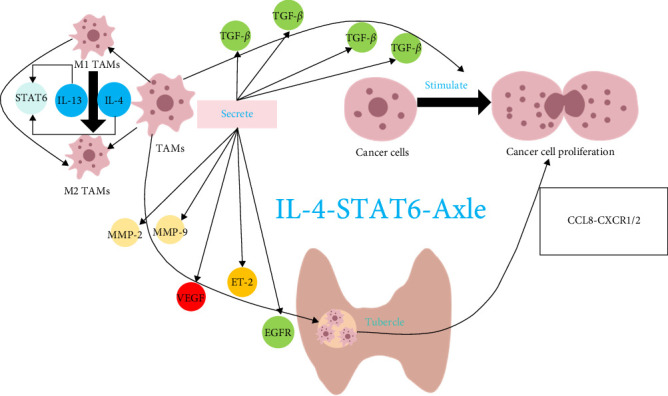
The influence of tumor-associated macrophages on the TIME.

Macrophages are important orchestrators in the human immune system that present exogenous antigens and catalyze the triggering and augmentation of the adaptive immune response (Figure 4). However, TAMs have been found to have a paradoxical role in that they do not directly destroy tumor cells, but actively foster tumor growth, invasion, and metastasis [57]. Within the densely populated landscape of the TME, macrophages are dominant, migrating to tumor foci and contributing to the reshaping of the tumor matrix, immunosuppression, and angiogenesis by secretion of an array of cytokines that promote tumor progression. The mechanism underpinning the protumorigenic effect of TAMs potentially involves suppression of antitumor functionality by transforming growth factor-beta (TGF-*β*), promoting migration, and recruiting a substantial number of TAMs within the tumor milieu, thereby promoting progression of the tumor via activation of the CXCL8-CXCR1/2 signaling axis [58]. TAMs are dichotomously classified into M1 and M2 phenotypes, each assuming prominence at distinct tumor stages. M1-like TAMs not only directly eliminate tumor cells but also play a crucial role in the early stages of cancer by activating other immune cells. These macrophages mediate antitumor immunity by stimulating both innate and adaptive lymphocytes. M1-like TAMs secrete or induce the production of immune modulators such as IL-6, IL-12, and TNF-*α*, which potentiate the antitumor activity of T cells and NK cells [59]. Furthermore, M1-like TAMs enhance the function of APCs, thereby amplifying the overall antitumor immune response [60]. In addition to their immunomodulatory effects, M1-like TAMs possess intrinsic cytotoxic potential, inducing tumor cell death, vascular damage, and tumor necrosis primarily through cytotoxicity and autophagy-mediated mechanisms [61]. M1-like TAMs predominate during the nascent phase but are gradually replaced by M2-like TAMs in the intermediary and advanced stages. Notably, the transition from the M1 to the M2 macrophage phenotype aligns with tumor progression.

M2-like TAMs are generally linked to poor prognosis, primarily due to their association with anti-inflammatory responses, tumor promotion, enhanced vascular survival, and increased tumor invasion [62]. During tumor invasion and migration, malignant cells release matrix metalloproteinases (e.g., MMP-2 and MMP-9), which breach the basement membrane and pave the way for metastatic dissemination [63]. Factors such as vascular endothelial growth factor (VEGF) and endothelin-2 (ET-2) have been implicated in angiogenesis, alongside secretion of multiple growth factors, including the epidermal growth factor receptor (EGFR) family of ligands, which increase the proliferation and migratory capacity of tumor cells [64]. Within the context of HT, polarization of TAMs towards the M2 phenotype is triggered by heightened expression of the cytokines IL-4 and IL-13 [65]. These cytokines exert their influence through signal transducers and transcription activator 6 (STAT6) proteins, thereby implicating activation of IL-4 and IL-13 as potential drivers of the pathogenesis and progression of HT [66]. This paradigm lends credence to the notion that HT has a modulatory effect on development of PTC by orchestrating the TIME and fostering polarization of M2-like TAMs via the IL-4-STAT6 axis [13]. In the presence of both PTC and HT, M2-like TAMs have an important role in promoting tumor progression and facilitating lymph node metastasis [67]. Notably, while the abundance of TAMs in PTC with concomitant HT remains substantial, the proportion of M2-like TAMs may diminish and the M1-type population could rise. This shift is associated with an improved clinical prognosis in cases of PTC concomitant with HT. Nonetheless, the precise mechanisms underlying the phenotypic changes in M2-like TAMs within the context of PTC and HT require further elucidation.

### 4.3. Effect of CAFs on the TIME

**Figure 5:**
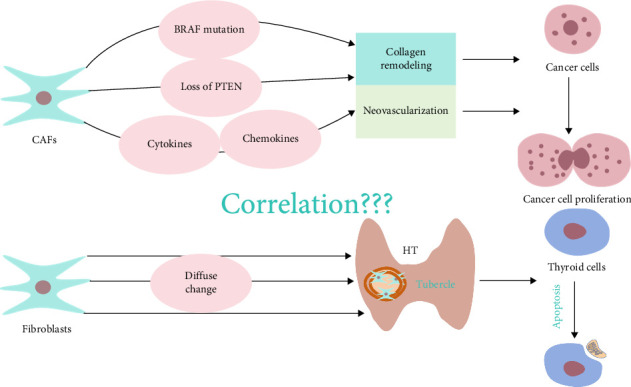
The influence of cancer-associated fibroblasts on the TIME.

CAFs, a subset of fibroblasts prevalent in both primary and metastatic tumors, have attracted substantial attention owing to their important role in initiation, progression, and metastasis of tumors [68]. Recent investigations have identified a subset of CAFs characterized by BRAF-like lesions with heightened aggressiveness [69]. Other studies have delineated how fibroblasts within the thyroid TME that harbor BRAF V600E mutation and PTEN deletion contribute to tumor progression by remodeling of collagen within the TME [70]. CAFs coordinate an array of tumorigenic processes, including angiogenesis, proliferation, survival, migration, and invasion of tumor cells, by modulating the production of antitumor immune cytokines and chemokines, thereby worsening PTC. Notably, heightened expression of CAFs has emerged as an indicator of adverse clinicopathological features in PTC, prompting discussion concerning their potential utility as biomarkers and therapeutic targets in cancer immunotherapy [40]. HT is a diffuse pathological process precipitated by a confluence of destruction of epithelial cells, infiltration of lymphoid cells, and fibrosis. Thyroid tissue from patients with HT is characterized by an abundance of lymphocytes that congregate at sites of follicular destruction and are encircled by connective tissue bands and clusters of fibroblasts. The pronounced presence of fibroblasts and collagen fibers within HT-affected thyroid tissue impairs substance exchange between thyroid cells and the capillary lumen, thereby increasing apoptosis of thyroid cells [71]. In the context of PTC concomitant with HT, the potential influence of CAFs on development of PTC and the precise mechanistic underpinnings warrant thorough investigation (Figure 5).

### 4.4. Influence of Related Factors on the TIME

**Figure 6:**
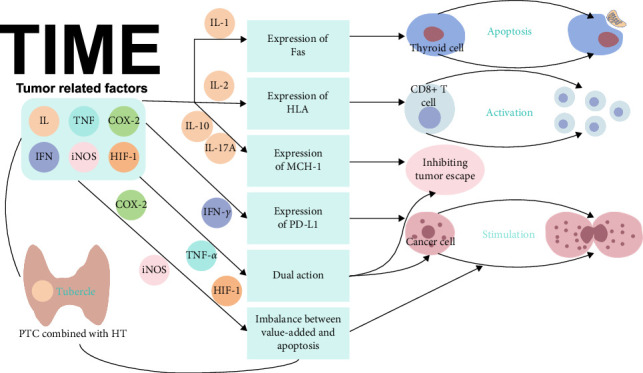
The influence of related factors on the TIME.

Over the past decade, TME-related factors and their mechanisms have emerged as a focal point in oncology research. Tumor cells have the capacity to recruit Tregs by creating an immunosuppressive microenvironment via secretion of specific factors [72], thereby promoting tumor progression. Concurrently, factors secreted by tumor-infiltrating immune cells contribute to an antitumor immune response (Figure 6).

ILs, which are integral to the TME, have a significant influence on tumor progression. Kimura et al. [73] demonstrated that IL-1 released by tumor-infiltrating lymphocytes can impede proliferation of thyroid cancer cells. Furthermore, Giodarno et al. [74] revealed that elevated IL-1 levels within the TIME of PTC concurrent with HT induce expression of Fas, triggering apoptosis in thyroid cells. In HT, this phenomenon may involve Fas and its ligands, whereby follicular cells promote destruction of tumor cells via Fas-mediated apoptosis pathways [75]. Fas activation regulates immune responses, tissue homeostasis, and immune clearance of viruses and tumor cells. Consequently, the beneficial impact of HT on PTC relates to eradication of tumor cells via humoral and cytotoxic T-cell-mediated immune mechanisms. Moreover, IL-2 can activate TME T-cells in thyroid cancer by augmenting human leukocyte antigen class I (HLA-I) molecule expression and tumor antigen presentation [75]. The heightened IL-2 levels in the TIME of PTC accompanied by HT can foster infiltration of CD8 + T cells, thereby boosting the anti-tumor effect. Furthermore, Luo et al. [76] noted an increase in IL-10 in patients with PTC and HT, which promoted remodeling of major histocompatibility complex class I (MHC-I) molecules and impeded tumor immune evasion.

IFN-*γ* has emerged as a prominent player in impairment of tumor immunity, notably by upregulating expression of programmed cell death ligand 1 (PD-L1) [77]. The activated PD-1/PD-L1 axis has been implicated in immune evasion by various malignancies, including PTC [78]. Experiments have identified this phenomenon during culture of PTC cells and peripheral blood lymphocytes subsequent to pretreatment with IL-17A. In vitro stimulation with IL-17A has been shown to augment expression of MHC I in PTC cells and to enhance T cell activation and expression of IL-2, while diminishing expression of IFN-*γ* and the PD-1 receptor. In this intricate process, IL-17A has an important role in thwarting tumor immune evasion. In summary, HT causes reduced expression of IFN-*γ* and PD-1 and heightened expression of IL-17A and MHC I in patients with PTC. There is some experimental evidence highlighting the potential of HT to impede tumor progression by modulating the expression levels of IFN-*γ*, PD-1, IL-17A, and MHC I. Notably, IL-2, IL-10, and IL-17A have been implicated in inhibiting immune escape mechanisms.

Alterations in TNF levels have considerable importance in the progression of PTC. TNF-*α* has a dichotomous impact on tumor evolution. In low concentrations, TNF-*α* is implicated in promotion of angiogenesis and facilitating tumor cell metastasis, whereas elevated levels of TNF-*α* may have antitumor effects [79]. Zhang et al. [80] demonstrated that TNF-*α* is significantly elevated in the serum of HT patients with PTC, suggesting that TNF-*α* could serve as a potential prognostic biomarker for postoperative outcomes in this patient population. TNF is recognized as a pivotal cytokine that is a bridge between inflammation and cancer.

Inflammatory mediators, including cyclooxygenase-2 (COX-2), inducible nitric oxide synthase (iNOS), and hypoxia-inducible factor-1 (HIF-1), have an important role in tumor progression. COX-2 is an inflammatory enzyme that is released into the TME by CAFs, TAMs, and malignant cells. The available evidence suggests that non-steroidal anti-inflammatory drugs targeting COX-2 activity can attenuate the incidence of certain cancers [81]. Notably, expression of COX-2 is heightened in PTC accompanied by HT, disrupting the balance between cell proliferation and apoptosis and influencing tumor initiation, progression, invasion, and metastasis [82]. Immunohistochemical investigations have identified markedly elevated COX-2 levels in PTC with HT, indicating a predisposition of patients with HT to develop PTC. iNOS serves as a downstream effector of diverse inflammatory cues and can be induced by other inflammatory factors, leading to excessive production of nitric oxide. Accumulation of nitric oxide can damage DNA and cause P53 mutations. Furthermore, induction of iNOS increases expression of COX-2 [83]. HIF-1, which is activated under hypoxic conditions, augments glycolytic activity in tumor cells and secretion of VEGF, promoting tumor progression [81]. In HT, inflammatory cascades and associated mediators modulate tumor progression, heightening the likelihood of neoplastic transformation when compared with HT-free scenarios.

Although many experimental studies have shown a complex immunological link between HT and PTC, the specific mechanism remains unclear. At present, most researchers agree that the prognosis of PTC with HT is better than that of PTC alone.

## 5. Conclusions

HT and PTC often occur concomitantly and have numerous shared pathogenic mechanisms and interactions. Immunologically, HT may contribute to the onset of PTC via intricate immunological pathways that are stimulated by prolonged inflammatory infiltration. The impact of HT on PTC is paradoxical. While HT can foster onset and progression of thyroid malignancy, tumor cells shape the TME in a reciprocal manner by releasing pertinent factors. Cytokines and chemical mediators released by tumor cells and infiltrating immune cells within the TME drive proliferation, migration, angiogenesis, and metastasis of tumor cells through diverse mechanisms. These factors also help tumor cells to evade immune surveillance, thereby promoting tumor progression. Coordination of the dynamics of the TIME and polarization of M2 macrophages via the IL-4-STAT6 axis facilitate carcinogenesis in patients with HT. At the same time, factors such as COX-2 and low levels of TNF-*α*, iNOS, and HIF-1 promote progression of HT towards PTC. Remarkably, the prognosis is better in patients with both PTC and HT than in those with PTC alone and may be linked to the abundance of CD8+ T cells. Patients who have PTC concomitant with HT have an elevated CD8+ T-cell count. The temporal dynamics and spatial distribution of these cells dictate the immunophenotype of PTC, and the prognosis is more favorable for the immunoinflammatory subtype. Furthermore, certain antitumor factors, including IL-2, IL-10, and IL-17A, mitigate tumor immune evasion, contributing to improved outcomes.

In recent years, the question of whether HT increases the incidence of PTC has sparked considerable debate. As researchers delve deeper into the TME and immunology, the important role of the immune milieu in the onset and progression of PTC has become increasingly evident. Immunological alterations have gradually come to be recognized as one of the main hallmarks of tumors. Immunotherapy has emerged as a focal point in cancer research, particularly with regard to anticancer immunotherapy and immune checkpoint inhibitors. These therapies exert their effects by stimulating lymphocyte activation against cancer cells, suppressing immunosuppressive signals, and thereby, encouraging a sustained antitumor response. The findings of preclinical and preliminary clinical investigations of monoclonal antibodies that target the PD-1/PD-L1 axis in conjunction with BRAF inhibitors have been promising. Furthermore, the preliminary outcomes of several ongoing experimental and clinical trials hold promise for development of novel targeted immunotherapies for advanced thyroid cancer. Advances in and maturation of immunotherapy have charted a fresh course for treatment of cancer. However, the association between HT and PTC, as well as the immunological mechanism underlying their convergence, remain unclear. The journey toward immunotherapy for HT with PTC is still fraught with challenges and requires further research.

## Figures and Tables

**Table 1:** The relationship between PTC and HT.

Clinicopathological profile	Conclusion	References
Age and sex	Compared with PTC alone, PTC patients with HT were younger and more likely to be female	[14, 18]
Preoperative thyroid function and antibodies	Preoperative serum levels of TGAb, TPOAb, and TSH were generally higher in patients with PTC with HT	[18]
Pathological stage	Patients with PTC accompanied by HT had earlier TNM stage	[19]
Maximum tumor diameter	HT has the effect of limiting the growth of tumor volume, but the tumor changes are multifocal	[20]
Lymph node metastasis	There was a negative correlation between HT and PTC and central lymph node metastasis	[20, 21]
Incidence of PTC	HT may increase the incidence of differentiated thyroid carcinoma (DTC)	[7, 9–12, 22]
Prognosis	HT was positively correlated with overall survival in PTC patients, suggesting that PTC combined with HT had a better prognosis	[9, 10, 19, 23–27]

## Data Availability

The data that support the findings of this study are available from the corresponding author upon reasonable request.
